# The Role of Alpha Oscillations among the Main Neuropsychiatric Disorders in the Adult and Developing Human Brain: Evidence from the Last 10 Years of Research

**DOI:** 10.3390/biomedicines10123189

**Published:** 2022-12-08

**Authors:** Giuseppe Ippolito, Riccardo Bertaccini, Luca Tarasi, Francesco Di Gregorio, Jelena Trajkovic, Simone Battaglia, Vincenzo Romei

**Affiliations:** 1 Centro Studi e Ricerche in Neuroscienze Cognitive, Dipartimento di Psicologia, Alma Mater Studiorum—Università di Bologna, 47521 Cesena, Italy; 2 UO Medicina Riabilitativa e Neuroriabilitazione, Azienda Unità Sanitaria Locale, 40133 Bologna, Italy; 3 Dipartimento di Psicologia, Università di Torino, 10124 Torino, Italy

**Keywords:** Schizophrenia Spectrum Disorder (SSD), Major Depressive Disorder (MDD), Attention Deficit Hyperactivity Disorder (ADHD), Autistic Spectrum Disorder (ASD), neuropsychiatric disorders, EEG, alpha oscillations, alpha frequency, alpha amplitude, connectivity

## Abstract

Alpha oscillations (7–13 Hz) are the dominant rhythm in both the resting and active brain. Accordingly, translational research has provided evidence for the involvement of aberrant alpha activity in the onset of symptomatological features underlying syndromes such as autism, schizophrenia, major depression, and Attention Deficit and Hyperactivity Disorder (ADHD). However, findings on the matter are difficult to reconcile due to the variety of paradigms, analyses, and clinical phenotypes at play, not to mention recent technical and methodological advances in this domain. Herein, we seek to address this issue by reviewing the literature gathered on this topic over the last ten years. For each neuropsychiatric disorder, a dedicated section will be provided, containing a concise account of the current models proposing characteristic alterations of alpha rhythms as a core mechanism to trigger the associated symptomatology, as well as a summary of the most relevant studies and scientific contributions issued throughout the last decade. We conclude with some advice and recommendations that might improve future inquiries within this field.

## 1. Introduction

Neuropsychiatric disorders are currently one of the most important sanitary emergences to watch for in high- and middle-income countries. Recent data collected on the European population [1] reports that approximately 165 million people are affected each year by mental disorders, and it is estimated that more than 50% of the general population in middle- and high-income countries will suffer from at least one mental disorder at some point in their lives. Some pieces of evidence [2] suggest that these results may be an underestimation, which would further increase these numbers. This would have enormous consequences from an economic perspective, and in terms of years of life lived with disability. This burden involves children as well, especially those from economically developed countries, even though an increase in other countries is soon expected [3]. Nonetheless, despite its relevance, it is still difficult to make a proper diagnosis because of the vast range of manifestations and overlap among the symptoms that may exist even inside the same cultural group, making these symptoms somewhat elusive to behavioral inspection [4,5,6]. In fact, despite the availability of numerous disorder assessment scales, the misdiagnosis rates are still high, with some reports indicating that over one third of patients are misdiagnosed [7,8,9,10]. This has obvious consequences on the patient’s well-being. Therefore, it is crucial to find a more reliable way of accurately making differential diagnoses, thus enabling prompt and proper intervention.

To this aim, various efforts have been put towards identifying neural markers capable of discriminating between neuropsychiatric disorders, treatment response, and the outcome prediction [11,12]. The electroencephalogram (EEG) has been very helpful, since it allows the brain electrical activity to be recorded in a totally non-invasive manner, with a relative fast montage and a high temporal precision [13,14]. For these reasons, it has been widely used to assess brain function in both the healthy and pathological populations. Indeed, the EEG is actually used in several clinical settings with diagnostic and prognostic purposes [15,16]. In the research field, it is commonly used to link a modulation in its signal during a task execution (ERP, event related potential) to a specific cognitive process [17,18]. Further, it is possible to investigate the cognitive functions through the analysis of the brain’s oscillatory activity.

### The Use of Alpha Rhythm for the Study of the Cognitive Functioning

Since Hans Berger’s [19] early studies, it has become evident that oscillatory patterns can be extracted from the brain’s electrophysiological signal, resulting from the nearly simultaneous firing of large ensembles of neurons. This rhythmic activity has been differentiated into five main functional categories, according to its frequency [20,21]: delta (δ; 0.5–3 Hz), theta (θ; 4–7 Hz), alpha (α; 8–13 Hz), beta (β; 14–30 Hz), and gamma (γ; 30–50 Hz). Their power is progressively reduced by increasing frequency, with a 1/*f* ratio [22].

These rhythmic oscillations can be detected by analyzing the signal obtained from the spontaneous brain activity, or in response to a sensory stimulation, internally or externally driven [20]. Thus, these frequencies have been linked to sensory and cognitive aspects, such as perception, attention, memory, or even consciousness [23,24,25]. Consequently, their disruptions have been associated with alterations such as the ones occurring in brain lesions or neuropsychiatric disorders [20,26]. It follows that the brain’s oscillatory activity can possibly represent an electrophysiological marker of these conditions, in which, although there is a biological connotate, it is still difficult to make a diagnosis after a behavioral evaluation. In particular, alpha oscillations have been linked to several cognitive processes such as memory, attention, distractor suppression, or even language [25,27].

Alpha oscillations are the most prominent rhythm in the human electroencephalographic signal [28]. A significant modulation of the alpha amplitude after external or internal events is called event-related synchronization (or desynchronization) (ERS/ERD), and is ascribable to an increase (or a decrease) in the rhythmic activity of a large number of neurons. Alpha synchronization seems to have a role in maintaining an active and adaptive inhibitory mechanism during perceptual suppression of upcoming information [29]. This inhibitory mechanism is enacted through a reduction in the cortical excitability that lessens the processing capacity of a particular area which is irrelevant to the ongoing processing [30,31]. Therefore, a synchronization within the alpha band may be an electrophysiological correlate of an information suppression mechanism [32]. Moreover, alpha amplitude has an important role in predictive processes, since the ability to predict the identity [33] or even the probability of occurrence [34] of the incoming stimulus modulates alpha power, which plays a role in preparing the brain for forthcoming stimulus perception [35,36]. Indeed, in the perceptual decision-making domain, alpha power appears to drive choice bias, as alpha ERD correlates with the adoption of a liberal criterion, increased decision confidence, and visual awareness [37]. On the other hand, it has been causally demonstrated [38] that the frequency of the maximum power within the alpha band (i.e., individual alpha frequency, IAF) is a critical parameter in defining sensory accuracy. Indeed, reducing (or, otherwise, increasing) IAF would result in a worsening (or an improvement) of the individual’s accuracy level [39,40,41]. Altogether, these results highlight the functional role of alpha activity in perceptual and cognitive processes. Moreover, the oscillatory activity can be used as a connectivity index between two or more brain regions, comparing the synchronization in phase or in amplitude over the areas of interest [42]. This kind of information is particularly useful when considering the oscillatory activity within the alpha band, given the empirical evidence supporting its involvement in diverse cognitive functions.

It follows that the alpha-based indices can be used to assess cognitive functioning in a broad range of pathological conditions [43,44,45]. In particular, neuropsychiatric diseases seem to be associated with anatomical and functional changes in the brain architecture, including connectivity alterations [46,47]. For instance, a reduction in the alpha power over the occipital regions is reported in children with Attention Deficit Hyperactivity Disorder (ADHD) [48,49]. This alteration seems to interfere with the processing of relevant and irrelevant stimuli, contributing to the attentional deficits in ADHD patients. Therefore, the behavioral symptoms of these disorders seem to be accompanied by alterations in the alpha-band oscillatory activity. For this reason, alpha-based parameters have been useful for the diagnosis of diverse neuropsychiatric disorders, as highlighted by the number of publications linking anomalous alpha activity to these conditions. In particular, among the conditions included in the DSM-5, the literature mostly focused on disorders such as schizophrenia, depression, ADHD, and autism. This also emphasizes the relevance of alpha band parameters as potential neuromarkers when exploring the disorders’ expression. Thus, a better understanding of the role of alpha waves for the manifestation of the symptoms would help to better comprehend the disorder and its associated biotypes. This could allow for a proper diagnosis and help to build more effective treatments and interventions.

However, even if the EEG-derived indices are commonly used in the clinical practice, there is still a lack of agreement about the diagnostic accuracy of these measures. In fact, even if there are plenty of EEG indices available in the literature, the results are often conflicting, if not opposing [50]. This is mostly caused by the heterogeneity of the studies and the different pathological biotypes [51], instead of the fast technological and methodological advances which this field of research has witnessed in the last few years. Indeed, while sometimes these new indices confirm the previous results, other times, they do not. Thus, the aim of the present work is to examine the existing literature to find reliable indices that would support the operator during the diagnosis and intervention procedures. To better identify how these disorders differ at the neural level, we took into account both local and connectivity electrophysiological indices within the alpha band, in order to depict the issue from a broader perspective (see Table 1).

Hence, given the abundance of work referring specifically to alpha oscillations, we aimed to summarize the literature regarding the main neuropsychiatric disorders in both adult and developing age groups which have emerged over the last 10 years. This work’s objective is to give the reader an overall perspective on how the alpha-based indices can be useful when investigating neuropsychiatric conditions, to help isolate the core features that better depict them. This may help with finding more reliable diagnostic and prognostic indices for each condition. For this reason, we excluded from the current work articles including patients with multiple diagnoses, or investigating comorbidities. Furthermore, while inspecting the publications, we also excluded those investigating drug effects or animal studies, in order to focus specifically on those studies which could help to define a more concise view of these conditions.

## 2. Schizophrenia Spectrum Disorder (SSD)

Although over a hundred years have passed since Kraepelin’s pioneering works on dementia praecox, the nosographical profile of Schizophrenia Spectrum Disorder (SSD) still displays some core clinical features that place this disorder among the most debilitating psychiatric syndromes. These hallmark symptoms include psychotic-like manifestations, such as delusions and hallucinations (positive symptoms), amotivation, anhedonia, and social withdrawal (negative symptoms), along with severe deficits in the cognitive (i.e., attention, working memory [WM], executive functions) and perceptual domain [52].

Inquiries aiming to elucidate the pathophysiological mechanics behind the emergence of SSD have recently focused on providing an oscillatory account of the aforementioned impairments, implying that such deficiencies might be engendered by a systematic failure to temporally integrate local neural activities into large-scale networks [53,54]. These considerations substantiate a conceptual framework based on the notion of SSD as a disconnection syndrome, where an aberrant decrease (or increase) in cross-regional synchronization might be responsible for the behavioral abnormalities witnessed in SSD patients [55]. Accordingly, it has been proposed that this rhythmic dysconnectivity may elicit primary deficits at a cognitive and perceptual level, whereas the subsequent pathological attempts at their resolution should lead to the onset of positive symptomatology [53].

On a molecular level, such oscillatory alterations are construed as a by-product of a malfunctioning of neurotransmitters’ dynamics. For instance, changes in inhibitory mechanisms mediated by gamma-aminobutyric acid (GABA)ergic interneurons (especially parvalbumin-positive cells) and reduced efficiency of N-methyl-D-aspartate receptors (NMDAR) have been tied to the emergence of SSD symptoms, which has also been associated with dopaminergic disturbances in mesolimbic and mesocortical loci [56]. While the complex interplay between these different dysregulations has been suggested to mainly affect activity in the gamma band, mounting evidence garnered in the last few years points toward their involvement in the generation of alpha rhythms as well [57,58,59].

### 2.1. Resting State Data

A seemingly common finding in SSD patients is a slightly reduced parieto-occipital spontaneous alpha power (resulting in an increased cortical excitability) as compared to healthy individuals [60,61]. This kind of evidence has been replicated in several studies exploiting resting-state data, which showed a tendency toward a reduction in local alpha activity as measured via power spectral density (PSD, i.e., the signal’s power content versus frequency) not only posteriorly, but also in frontal and central regions [61,62,63,64]. Moreover, a direct link has been found between this decrease in alpha power at the parietal and left frontotemporal sites and the gravity of the positive SSD symptomatology [65,66]. Accordingly, treatments capitalizing on alpha-tuned transcranial alternating current stimulation (tACS), which were administered to patients experiencing auditory hallucinations, were proven to restore such imbalances by boosting resting alpha power, consequently reducing the severity of hallucinatory symptoms [67]. However, recent evidence appears to depict a more nuanced portrait of the matter. While some studies described no reduction in terms of posterior alpha activity in SSD patients [68,69,70], one reported higher parieto-central alpha power in medicated patients as compared to healthy controls [71]. Such an increase was also outlined by data collected in three additional papers, focusing on, rather than task-positive regions (i.e., neural areas more active during attention-demanding tasks), task-negative areas, which are known to increase their level of excitability at rest [72]. A globally heightened spectral power was reported in SSD individuals [73,74] when compared to controls, whereas in a similar work, the same abnormal increase was found with regard to an alpha component located over parietal and temporal areas largely overlapping with the posterior portions of the Default Mode Network (DMN) [75]. Interestingly, spectral inquiries carried out on DMN sites via EEG and magnetoencephalography (MEG) showed comparable results, namely increased alpha power in the medial prefrontal and posterior cingulate cortices (mPFC and PCC) of SSD patients [76,77].

Another oscillatory parameter that can be extrapolated from resting-state power analyses is IAF, namely the exact frequency within the alpha band at the maximum amplitude value. The existing literature suggests that a faster (rather than slower) IAF entails better perceptual acuity and efficiency [38,78], with individuals displaying pronounced schizotypal traits characterized by slower IAFs [79]. Findings gathered over the last decade unilaterally uphold previous knowledge, namely slower resting-state IAFs in SSD patients [68,69,70,73,77], with the degree of deceleration positively correlating with visuo-attentional performance and scorings at cognitive scales [80]. Furthermore, faster IAFs in SSD individuals undergoing multisession cognitive training was found to predict protocol outcome (i.e., responders vs. non-responders). Along this line of reasoning, it has also been found that occipital IAFs of patients affected by negative symptoms cycle more slowly than in healthy participants, while the opposite held for individuals with SSD displaying positive symptoms [81]. Accordingly, IAFs in both groups of patients correlated with the severity of symptomatology, as measured via behavioral scales [62].

At-rest connectivity metrics have also been largely exploited to gain some insights into the network architecture of SSD patients. Heightened synchronization within the alpha band has been reported in first-episode schizophrenic patients, with cortical hubs sited along frontocentral, occipital, and right temporo-parietal regions displaying the highest levels of interconnections, which was negatively correlated with scorings at cognitive scales [82]. In a similar study, alpha coherence parameters were also found to be enhanced both in an inter- and intra-hemispheric manner in SSD patients [83]. Notably, synchronization measures gathered from DMN nodes are in line with such results (i.e., similar or increased inter-areal alpha coupling in SSD) [75,76,84]. On the other hand, several studies provided a different perspective on the way alpha oscillations behave on a network level in SSD. Evidence for globally reduced alpha synchrony over frontal areas has been outlined by means of different indices, such as phase-locking value (PLV) and phase-lag index (PLI), in patients compared to healthy controls, with a concurrent increase in the information flow from occipital to anterior sites (and a decrease in the opposite direction) [85]. A study adopting non-negative matrix factorization, as well as both energy and entropy measures of connectivity, in individuals with SSD unveiled a general decrease in alpha-band coherence within four spread clusters centered on the bilateral cingulate, left temporal-parietal, precuneal-PCC, and right prefrontal cortices [86]. These patterns of reduced rhythmic interaction were reported to be associated with patients’ psychiatric symptoms. Another work uncovered attenuation of inter-hemispheric alpha connections in SSD (but not healthy individuals) at multiple sites (frontal, parietal, and temporal) [68]. A decrease in alpha-tuned inter-hemispheric anterior connectivity and frontoposterior cross-talking was also highlighted in two further studies adopting connectivity analyses [87,88].

**Figure 1:**
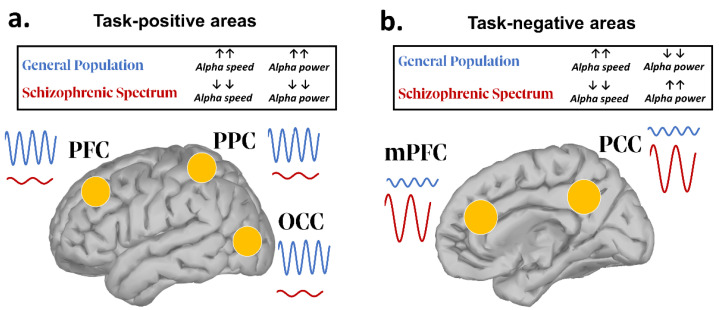
Graphical representation summarizing the main findings on resting-state alpha power in SSD (relative to healthy individuals). The left panel (**a**) depicts alpha oscillatory patterns relative to task-positive areas, while the right panel (**b**) depicts those relative to task-negative regions (largely overlapping with DMN’s nodes). Upward and downward arrows indicate respectively an increase or a decrease in alpha power or frequency speed. As for the former areas, alpha power is decreased, while the latter show an overall increase of such oscillatory index. PFC (prefrontal cortex); PPC (posterior parietal cortex); OCC (occipital cortex); mPFC (medial prefrontal cortex); PCC (posterior cingulate cortex).

To summarize, a significant slowdown of IAFs appears to be a core pathological feature characterizing SSD. As for oscillatory power, spontaneous alpha activity appears to be decreased and increased in SSD patients over, respectively, task-positive and task-negative regions (Figure 1). On a network level, dysregulations in both directions (higher vs. lower connectivity) in various neural clusters have been found, perhaps due to the different methodological approaches adopted (scalp- or source-based computations), or sample variability (first-episode vs. chronic or medicated vs. unmedicated patients).

### 2.2. Perceptual Impairments

Alpha oscillatory dynamics in SSD have been scrutinized in patients asked to perform perceptual tasks. Indeed, a plethora of deficits involving sensory processing has been reported in SSD, the severity of which has been hypothesized to trigger the onset of positive symptomatology [53,89].

Auditory hallucinations correspond to the most salient psychopathological manifestation in SSD. As such, defective mechanisms in the rhythmic signaling underlying auditory perception have been widely investigated to pinpoint some of the putative features driving this kind of hallucinations. Auditory steady-state response (i.e., the electrical response recorded from the auditory cortex to the entrainment induced by repetitive acoustic stimuli) paradigms have been frequently employed to probe the electrophysiological malfunctioning in SSD [90]. These studies reported aberrations in the low frequency bands (theta and alpha) in SSD (as compared to controls) during the task, such as lower evoked power, intertrial coherence (ITC), and increased theta–alpha phase–amplitude coupling (PAC) [90,91,92]. Similar tasks capitalizing on the presentation of multiple pairs of auditory stimuli yielded interesting results about whether and how alpha oscillations mediate perceptual impairment in auditory-related sensory gating. Specifically, evoked alpha power over posterior and midline sites has been shown to undergo a reduced suppression in response to the administered acoustic pairs in SSD as compared to controls [71,93,94], with the degree of (deficient) suppression being associated with GABAergic levels over frontocentral areas [95]. However, evidence for a demeaned alpha suppression over midline posterior areas, which tended toward a relative increase (i.e., more alpha suppression) over frontocentral sites, has also been reported in SSD [96]. Moreover, audio-verbal training was found to be effective in boosting such impaired alpha suppression in response to the second (but not the first) acoustic stimulus in each pair, leading to an oscillatory improvement that correlated with better scoring on verbal learning scales [97]. Impaired sensory gating was also shown to be accompanied by reduced frontocentral alpha ITC between the first and second auditory stimulus, which was inversely correlated with the negative symptoms assessed using a behavioral scale [98]. Consistent with this finding, a lower alpha-tuned inter-hemispheric coherence between temporal and parietal electrodes was outlined during the completion of a passive auditory task in patients with SSD experiencing auditory hallucinations (as compared to controls and SSD patients not affected by such symptoms). Likewise, aberrant rhythmic patterns within the alpha band have also been found through paradigms presenting a series of standard acoustic stimuli intermingled with deviant tones. Two studies showed that SSD patients exhibit lower ITC and evoked power than healthy participants over central regions in response to standard tones, with the magnitude of the power increase among patients correlating with verbal learning and working memory capacities [99,100]. However, higher evoked alpha power, after both standard and deviant stimuli, was also reported [101].

Overall, alpha rhythmic activity appears to be less reactive and susceptible to task-relevant suppression in patients engaged in perceptual tasks, which suggest both an over-increased power at rest and diminished control during the performance.

### 2.3. Cognitive Deficits

Impaired cognition is an additional feature enriching the already complex clinical phenotype of SSD. Subtle derangements at this level often tend to occur many years before the onset of psychotic symptoms, fueling the idea that delusions and hallucinations might represent a pathological attempt to make sense of erratic and vague information provided by deficient cognitive mechanisms [53]. While it remains to be clarified whether these deficits result from a disruption of lower-level perceptual mechanisms, various inquiries were set to further illuminate the oscillatory contributions to such phenomena.

The auditory oddball task has been among the most widely adopted paradigms to disentangle the neurophysiological correlates underpinning the way in which patients with SSD handle novel stimuli and cope with irrelevant information. For instance, SSD patients and individuals at a high risk of developing psychosis were found to display a reduced posterior alpha ERD as compared to healthy controls in response to target stimuli [102,103]. On the other hand, a similar study capitalizing on MEG recordings reported a significant decrease in alpha ERS over occipital and posterior midline regions [104]. Transcranial magnetic stimulation (TMS) treatments administered along the left frontoparietal axis to restore these disbalances showed that, in most SSD patients, an increase in task-related alpha power (recorded after, as compared to before, the TMS protocol) occurred in response to both rare and frequent stimuli. This degree of spectral increase also displayed a slight association with improvements in positive and negative symptomatology, as assessed via behavioral scales [105].

Alpha ERD/ERS dysregulations in SSD have been explored even via working memory (WM) paradigms. Indeed, individuals with SSD exhibit a reduced contralateral alpha suppression (ERD-like response) in trials with higher cognitive loads. This aberrant reduction correlated with worse WM performance both between (lower SSD relative to controls) and within (lower SSD with less alpha suppression relative to those displaying greater reduction) groups. In the latter case, a significant relationship with diminished contralateral ERD and the psychiatric symptoms was also uncovered [106]. Furthermore, a comparable reduction in contralateral alpha ERD, coupled with impaired behavioral performance, was reported in SSD in two similar studies [107,108], while weaker inter-areal connectivity within the alpha range has been outlined between occipital regions and several temporo-parietal and frontal clusters (the magnitude of which was associated with the severity of positive symptoms) [109].

Maladaptive alpha dynamics also appear to arise in SSD individuals instantiating inhibition-related mechanisms (or a release from inhibition). Patients with SSD showed higher alpha ERD over frontal and temporal sites during the inhibitory task [110]. Conversely, reduced alpha suppression over sensorimotor cortices was also found in SSD during incongruent trials of a Stroop task (in association with slower reaction times) [111].

Lastly, mounting evidence suggests that a disruption in alpha dynamics might also be involved in social cognition impairments (i.e., poor empathy judgment and mentalizing skills, along with pathological social withdrawal) [112]. Specifically, SSD patients engaged in a facial emotion recognition task displayed lower alpha power and higher connectivity within the alpha band over frontocentral sites, together with lower accuracy in recognizing both happy and fearful emotional expressions [113]. When performing an ecological face-to-face interaction with a confederate, the SSD group displayed an alpha connectivity increase undetected in healthy controls during the more affiliative task condition (*closeness* condition). Such an increase was found to be positively correlated with negative symptoms [114]. Aberrant modulation of alpha power in SSD was also noted during the Ultimatum Game, a social decision-making task involving a fair split of a sum of money between other humans or a computer [115]. Patients displayed a more robust upregulation of alpha power over midfrontal spots during the anticipation phase when playing with a computer vs. a human agent. This power difference was proven to be negatively correlated with positive symptoms [116]. Moreover, in a self-referential memory task, SSD displayed higher scores when presented with self-related items (as compared to neutral or other-related items), which was paralleled by a demeaned alpha ERS over the midline and right frontocentral regions during the encoding phase, and a massive reduction (relative to healthy subjects) in long-range alpha synchronization across multiple cortical electrodes [117].

Altogether, these findings (Table 2) resemble those concerning perceptual processing, namely a pervasive dysregulation in the way alpha oscillations instantiate phasic fluctuations in event-related cortical excitability (ERD vs. ERS). Regarding connectivity metrics, the results are more interspersed, even though they seem to point toward a reduction in long-range alpha coherence across different cognitive tasks, suggesting a bioelectrical disruption in the way higher-level neuronal firing modulates the activity of lower areas.

## 3. Major Depressive Disorder (MDD)

Major depression has been described as a psychiatric syndrome whose core features entail persistently low mood and anhedonia, coupled with sleep and psychomotor disturbances, fatigue, or loss of energy. Alterations in the affective domain might also involve feelings of worthlessness or guilt and suicidal thoughts, suggesting a pervasive tendency toward negative self-referential thinking and ruminations. In addition, subclinical cognitive deficits (i.e., diminished ability to concentrate, impaired attentional and memory functioning) have often been reported in patients suffering from this disorder [52].

**Figure 2:**
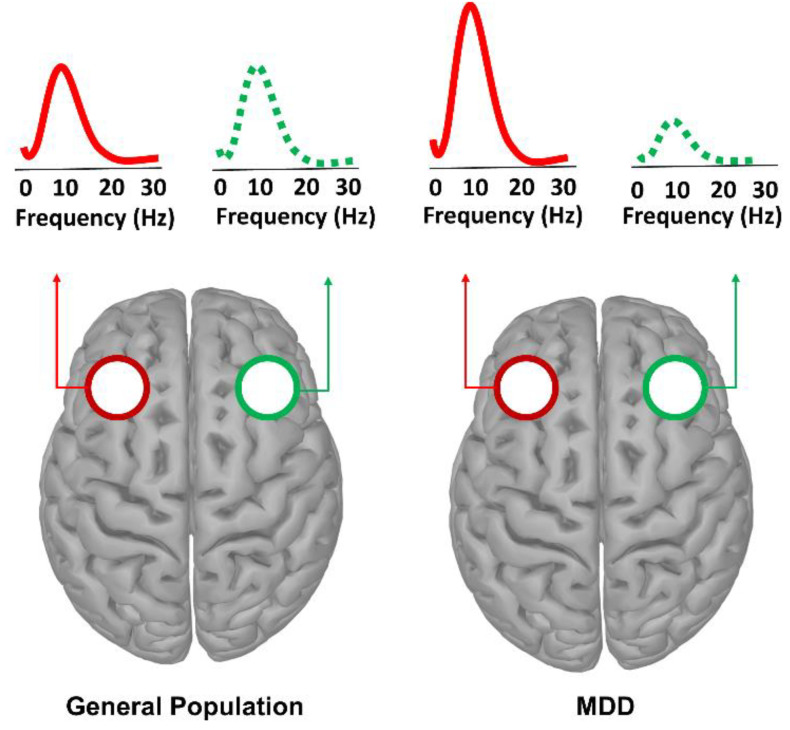
Schematic depiction of FFA patterns witnessed in depressive patients. Relative to healthy individuals (left panel), MDD patients (right panel) display higher relative alpha power over the left (vs. right) frontal cortex.

Oscillatory insights into the pathophysiological dynamics underlying MDD suggest that individuals diagnosed with this disorder exhibit rhythmic aberrations, encompassing lower frequency bands, which is likely due to alterations occurring at cortical and subcortical loops that coalesce into a neuropathological phenotype known as thalamocortical dysrhythmia [64,118,119]. Consistent with this notion, MDD patients display higher alpha power levels over the left (relative to the right) frontal lobe, an oscillatory pattern called frontal alpha asymmetry (FAA; Figure 2) [120]. Given the inverse relationship between alpha rhythms and cortical excitability, this electrophysiological abnormality has been framed within the approach–withdrawal model [121], where an increased activation of the right vs. left frontal cortices has been thought to occur in individuals more prone to behavioral withdrawal vs. approach (i.e., proneness toward a negative vs. positive affective style). As such, FAA has been deemed as one of the most reliable biomarkers (although for a different account, see [122,123,124]) indexing MDD-related affective asymmetries responsible for symptoms such as hopelessness or helplessness and anhedonia [118,120,125].

Aside from FAA, while it has been recently proposed that MDD might be also characterized by interhemispheric asymmetries over posterior sites (i.e., posterior, or parietal, alpha asymmetry, PAA) [118], interareal communication seems to be altered as well. For instance, MDD patients displayed higher alpha-band coherence within the DMN and between anterior midline areas and the frontoparietal network [126,127]. These aberrant patterns of interregional cross-talking are assumed to underlie many functional dysregulations, putatively resulting in ruminative thoughts, increased self-focus, and a reduced ability to concentrate and properly deploy attentional resources [127].

Thus, a general disruption in alpha rhythms, occurring on several different levels, seems to crucially contribute to the pathogenic mechanics behind MDD syndrome and some of its associated affective and cognitive symptoms.

### 3.1. Resting State Data

Much of the resting-state literature gathered over the last decade has been devoted to the investigation of FAA, with the aim of affirming its role as a neuromarker able to provide an early diagnosis in individuals at risk, but also to anticipate the clinical outcomes. A stronger activation in the alpha band of the right frontal cortex in MDD was replicated in several studies [128,129,130,131,132,133,134,135], some of them showing a robust relationship between the degree of FAA and scores on depression behavioral scales [136,137]. FAA has also been proven to predict treatment effectiveness. For instance, FAA seems to display a negative correlation with sensitivity to specific psychotherapeutic protocols [138], while bifrontal alpha-tuned tACS was capable of reducing both alpha power over the left prefrontal cortex (i.e., asymmetry decrease) and the depressive symptoms [139,140].

Lateralized patterns of rhythmic activation have been reported over posterior sites as well. Although not as straightforward as FAA, PAA appears to be a recurrent oscillatory feature of MDD [118]. Specifically, alpha power was found to be enhanced over the left parietal cortex of female patients suffering from MDD [131], whereas a sample of depressed adolescents tested in a different study displayed an opposite lateralization trend, which was associated with rumination and anhedonia symptoms [141]. Further, MDD patients less sensitive to deep-brain stimulation treatments seem to show an increased parietal alpha activity over the left hemisphere, with the magnitude of PAA negatively correlating with behavioral depression scores [142]. In addition, anodal transcranial direct-current stimulation (tDCS) over the dorsomedial PFC was shown to induce a greater reduction in anxiety scores in patients displaying lower and higher baseline alpha power, as recorded from, respectively, the left parietal lobe and the precuneus [143].

Apart from oscillatory asymmetries, canonical spectral aberrations within the alpha band have also been described in patients exhibiting suicidal ideations [144]. Higher parieto-central alpha power was also found to characterize elders suffering from late-life depression [145], and to better discriminate MDD individuals from healthy participants [146]. Moreover, a positive clinical outcome in depressed patients has been associated with a decrease in frontal alpha power triggered by 10-Hz repetitive TMS protocols and electroconvulsive therapy [147,148]. However, some studies also reported lower posterior oscillatory power within the alpha band in MDD [149,150], the degree of which was linked with depression severity and attentional impairments [151,152]. Conversely, symptomatologic improvements in MDD have been proven to correlate with frontal and midline alpha power increases stemming from alpha-tuned repetitive TMS protocols [153,154].

Resting-state paradigms have also been exploited to address whether and how MDD might alter rhythmic connectivity dynamics. To begin with, proneness to brooding rumination appeared to go along with reduced alpha–gamma PAC over posterior brain areas [155], and global reduction in functional connectivity within the alpha band has been proven to correlate with depression severity [156]. In a network-based study adopting both nodal and global measurements, lower alpha connectivity was uncovered in MDD. Additional analyses also revealed decreased nodal clustering in several cortical hubs spread over frontal regions, as well as the temporal lobe and the visual cortex [157]. Multi-layer analyses run on MEG data to explore interareal oscillatory cross-talking showed similar results, namely a decrease in alpha connectivity in depressed participants [158]. Conversely, alpha-tuned repetitive TMS protocols were shown to ignite an increase in connectivity in MDD, displaying symptom improvements [159]. Still, these results are challenged by plenty of data suggesting an opposite (positive) relationship between levels of alpha connectivity and depression severity. Increased interhemispheric (central) and intrahemispheric (right frontocentral) alpha coherence was found to discriminate depressed individuals from those diagnosed with bipolar disorder [160]. Similarly, MDD displays higher alpha coupling, linking frontopolar loci to the temporal and parieto-occipital regions [161]. Moreover, depressed patients also displayed increased cross-regional alpha connectivity between the ventromedial PFC and both the left mPFC and left dlPFC, as well as between the subgenual anterior cingulate cortex (ACC) and the left dlPFC [162,163]. Furthermore, positive outcomes yielded by antidepressant-based treatments have been associated with weaker alpha oscillatory connectivity bridging the insular cortex to the rostral ACC [164], whereas the magnitude of the TMS-driven decrease in spectral correlation metrics relative to the left hemisphere was shown to be correlated with clinical ameliorations [165].

Taken together, resting-state data concur in assigning FAA (and to a lesser degree PAA) a pathophysiological role in the emergence of MDD. As for more generic impairments in oscillatory power and connectivity, the aforementioned patterns appear more difficult to interpret. These controversial findings might be construed as the result of heterogeneous clinical samples in terms of age or gender, not to mention the different frequency bins (within the canonical alpha band) adopted throughout the analyses. Lastly, a scenario where MDD might exhibit diverging rhythmic phenotypes should not be ruled out; this jeopardizes a unified interpretation of the matter.

### 3.2. Cognition, MDD and Alpha Rhythms

Oscillatory fingerprints underpinning impaired alpha activity in MDD are not restricted to the resting brain. Electrophysiological investigations during cognitive tasks are consistent with the notion that cortical circuits responsible for attentional deployment, memory storage, and executive functions display demeaned activation in MDD [127].

For instance, during the auditory oddball task, MDD patients who exhibited lower IAFs and alpha power at rest were shown to upregulate such parameters during tasks in response to deviant stimuli [150], along with increased event-related alpha phase synchronization between frontocentral and parieto-occipital electrodes in response to target stimuli during a visual oddball paradigm [166].

With regard to the WM domain, MDD patients displayed poorer behavioral performance on a Sternberg task, which was associated with decreased posterior alpha power during the retention period [167,168]. Moreover, lower increases and decreases in alpha phase synchronization within, respectively, the frontoparietal route and occipito-central cortical clusters have been reported in MDD during the n-back task [169].

Altered patterns of alpha activity appear to also underlie impairments in inhibitory mechanisms, as measured using Go/No-Go tasks. For instance, both reaction times and accuracy rates were reported to be significantly lower in depressed patients displaying suicidal behavior. These data were linked to abnormally high alpha power levels recorded from the ventromedial PFC and ACC during, respectively, Go and No-Go trials [170]. Further, mindfulness-based therapeutic protocols have been reported to enhance task-related alpha ERD and left frontoparietal coherence during the same task, with the degree of oscillatory realignment shown to correlate with clinical ameliorations [171].

Defective emotional processing is an additional symptomatological feature characterizing major depression, with disrupted alpha rhythm dynamics contributing to this pathological phenomenon. In a sample composed of MDD patients with and without dysphoria, higher bilateral frontal alpha power was reported in response to both pleasant and unpleasant emotional stimuli only in MDD patients with dysphoric symptoms [172]. During an emotion self-regulation study employing happy emotion induction training, a power decrease in the upper alpha band over the frontal and temporal sites was reported during the first session of the protocol. Intriguingly, changes in task-related FAA (asymmetry decrease) resulting from such self-regulation training were proven to correlate with scoring at the behavioral scales [173]. Similarly, bifrontal alpha tACS applied to MDD patients was reported to attenuate left alpha power, and thus FAA, during the passive viewing of emotionally positive images [139]. In addition, a weaker posterior alpha ERD was found in MDD patients engaged by WM items superimposed on negative emotional pictures, whereas the opposite pattern was witnessed when positive emotional pictures were presented in the background [174].

Biases in emotional processing have also been investigated through face recognition paradigms. For instance, FAA appeared to unfold in MDD in response to both happy and sad faces [175]. Moreover, alpha–gamma PAC unveiled an attenuated interplay between right-lateralized cortical (i.e., orbitofrontal cortex) and subcortical (i.e., thalamus and amygdala) neural nodes in non-responders to antidepressant treatments, which was likely to reflect abnormal sensitivity to negative emotional stimuli [170].

Lastly, impaired alpha activity has been proven to play a pivotal role in higher cognitive abilities. Patients with MDD were found to display FAA patterns when performing a reinforcement learning task, the occurrence of which has been deemed to underlie losses in approach-related motivation [176]. Reduced alpha power over the left hemisphere was also reported during a multi-stage decision-making task in MDD patients [134], while a neurofeedback training was able to boost alpha power as measured during the completion of a counting task over the ACC and parieto-occipital sites [177].

In brief, task-relative alpha dysregulations tend to be often witnessed in MDD. Aside from FAA, generic alterations in oscillatory power and inter-regional communication have been found to underpin different cognitive deficits. However, the direction of these alterations is still unclear (Table 3), and seemingly depends on each individual’s pathophysiological biotype, as well as the specific cognitive process.

## 4. Autism Spectrum Disorder (ASD)

Autism spectrum disorder (ASD) is a complex neurological and developmental disorder characterized by impairments in social cognition and interaction other than sensory and perceptual abnormalities [178,179]. The behavioral and cognitive symptoms associated with ASD are extremely heterogeneous, and the diagnosis is based on different patterns of behavior, including: (a) persistent deficits in social communication and social interaction, such as socio-emotional reciprocity, non-verbal communication, or difficulties adjusting behavior according to various social contexts; (b) restricted interests, repetitive patterns of behavior, such as stereotyped motor movements, inflexible adherence to routines, or unusually intense interests [179].

### 4.1. The Role of Alpha Oscillations in ASD’s Symtomatology

**Figure 3:**
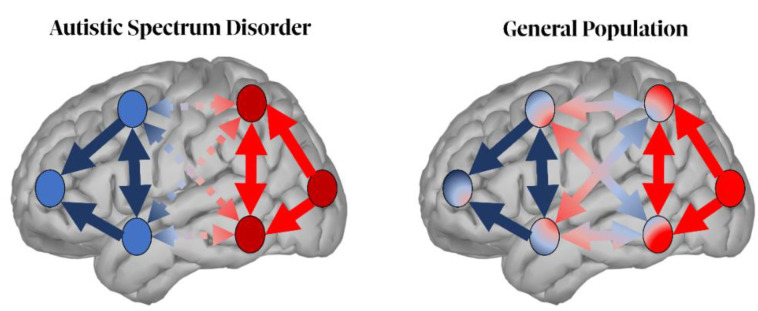
Imbalance in alpha connectivity in ASD relative to typically developing individuals. Persons with ASD show a local increase in connectivity in both posterior and anterior areas, while long-range connectivity between these two regions is reduced (dashed lines). Conversely, neurotypical individuals show the opposite pattern, resulting in a minor local integration (shaded circles).

Studies on the neural underpinnings of ASD show functional and anatomical alterations within the perceptual neural network [180,181,182]. ASD individuals showed alterations in long-range structural and functional connectivity (rather than in a local areas) [180,183,184,185]. Specifically, ASD individuals seem to display a reduction in interhemispheric long-range synchronization within the alpha band. Indeed, it has been demonstrated that ASD individuals have a reduction in the alpha phase coherence between temporal regions [181]. Other authors [186] showed that frontal alpha asymmetry in 6-month-old children correlates with ASD diagnosis at 24 months. Similarly, connectivity measures within the alpha band have also been linked to sensory symptoms assessed using behavioral scales [180]. These results point toward a decrease in long-range cross-talking, although additional evidence also reports an increase in short-range connectivity among the ASD population (Figure 3) [180,181,187]. In general, these connectivity patterns seem to significantly differ from the ones found in neurotypical individuals, reflecting an atypical brain network development in ASD. Specifically, while neurotypicals show a strengthening of long-range connections and a weakening of short-range ones with aging, the opposite tendency can be seen in ASD [188,189]. Furthermore, several studies based on connectivity measures [182,190,191] highlight the role of the directional interactions among brain areas in ASD. In more detail, ASD individuals show a prevalence of ascending connections from posterior to anterior areas, pointing to a tendency to convey more bottom-up information. Altogether, and consistent with previous literature [192,193], these pieces of evidence highlight electrophysiological abnormalities in ASD (i.e., reduced interhemispheric connectivity over temporal and frontal regions). Since these aberrant modulations appear at early stages of development, they tend to be accompanied by anatomical disturbances, including atypical axon numbers, synaptogenesis, and pruning [187,194]. Altogether, these alterations seem to significantly contribute to the perceptual deficits and clinical manifestations of ASD.

A large number of studies also associated brain oscillatory activity with ASD symptoms. Specifically, Machado and colleagues [195] investigated the role of PSD in sensory elaboration in ASD, using a visual and audio-visual passive task. They reported a reduction in alpha power and an increase in slow-delta, high-beta, and gamma bands. This seems to be mainly due to the role that the alpha phase plays for the integration of cortical information, supporting executive function and the response to sensorial stimuli via the modulation of neuronal excitability [196]. Coherently, Han et al. [180] reported that brain connectivity within the alpha band correlates with symptom severity in ASD, specifically with the “sensory stimuli and relating behavior” subscale of the Autism Behavior Checklist. This alteration in brain activity may be responsible for a disruption in the excitatory/inhibitory balance and, consequently, may affect the neural response, leading to biased behavioral responses to sensory stimuli. Such imbalance could partly explain the unusual interest in simple objects characterizing people with ASD [180,196]. In line with this evidence, studies addressing how ASD individuals allocate attentional resources have revealed abnormal processing of the upcoming stimuli. Specifically, during an intersensory attention task, in which the suppression of a distractor is relevant to reaching good performance levels, ASD individuals do not show the anticipatory power increase in the alpha band over parieto-occipital areas that is seen in neurotypical individuals before the distracting stimuli [197]. The lack of this preparatory activity has been associated with impaired performance due to the presence of task-irrelevant sensory information. Furthermore, people with ASD did not exhibit any posterior alpha desynchronization (with the resulting reduction in power) during the appearance of a relevant target, which was associated with impaired behavioral performance and increased ASD symptomatology [198]. Thus, these aberrant alpha power modulation patterns seem to be linked to impairments in perceptual suppression of irrelevant sensory information, contributing to difficulties in focusing on relevant stimuli [180,196].

Another EEG measure linked to ASD symptoms is the mu–alpha suppression, i.e., a reduction in the alpha power over sensorimotor areas during action execution or observation of actions and facial expressions [199]. Consistent with the mirror neurons hypothesis, mu suppression reflects the internal simulations of others’ actions, allowing one to better understand their intentions [200]. Several studies [201,202] have shown less consistent mu–alpha suppression in ASD, which is possibly related to social deficits.

In conclusion, due to its heterogeneous manifestations, ASD can be challenging to diagnose using behavioral scales. The findings described here indicate that some of its symptoms can be linked to specific neural alterations, thus legitimatizing the search for neural markers of ASD. Thus, finding an electrophysiological correlate of ASD would considerably support and ease the diagnostical evaluation.

### 4.2. EEG Indices for an Early ASD Diagnosis

Despite the significant number of electrophysiological measures available in the literature, there is still a lack of concordance regarding which index would best represent an anatomical marker of ASD to assist an early diagnosis [178,203]. In fact, several MEG and EEG studies have reported anomalies in one or more frequency bands in ASD, linking these indices to symptom severity. Yet, the results are often conflicting [50,204,205,206,207,208,209,210,211]. This seems to be attributable to the great discrepancies between experimental design and procedures, as well as sample discrepancies (e.g., some studies were conducted on children, while others on adults, some on persons with a low- or high-functioning profile, under or without medications, etc.) [204,212,213,214].

An EEG study from Matlis and colleagues [203], conducted on a large sample of children with ASD, showed a robust reduction in the peak alpha-ratio (i.e., reduced posterior to anterior power ratio) in persons with ASD. The authors also used this index as an electrophysiological marker for ASD, reaching high accuracy levels. These results suggest that ASD individuals may have higher power values over the anterior areas, which correlates with behavioral inhibition and sociability [215,216]. A recent study [188] used the EEG data obtained from the spontaneous brain activity in 3-month-old infants to predict a later ASD diagnosis. This study highlighted that the best predictors of a later ASD diagnosis at 18 months are a lower frontal and a higher fronto-temporal connectivity. These results are consistent with the literature indicating the presence of hypoconnectivity within the frontal regions, which is in line with the executive and social difficulties among the ASD population [217,218]. Similarly, Orekhova et al. [219] demonstrated that high-risk 14-month-old infants later diagnosed with ASD showed higher alpha-based connectivity over the fronto-central areas. These results are in line with the aforementioned studies [187,220,221] suggesting that ASD individuals are associated with a shift from early white matter maturation during infancy to hypoconnectivity with aging.

Altogether, these data suggest the presence of an excitation–inhibition unbalance in the neural excitability in persons with ASD [178,222,223]. This feature could be responsible for a modified signal-to-noise ratio, resulting in an altered sensorial experience. In fact, due to the abnormal levels of cortical excitability, these individuals could be characterized by hypo- or hyper-responsiveness to sensorial stimuli, thus affecting several cognitive and perceptive domains. Therefore, such electrophysiological alterations may underlie perceptual alterations, contributing to explanations for some of the distinctive symptoms of ASD. The gamma band has been demonstrated to have a key role in these dynamics, given its involvement in the binding of perceptual information into one coherent whole via the integration of responses from near areas [224]. However, the results involving the measurement of the gamma frequency are often conflicting, since its large frequency spread makes it difficult to track the phase between brain areas [178]. To avoid these difficulties, it can be useful to use cross-coupling indices, in which the phase of a lower frequency oscillation in one area has been shown to modulate the amplitude of a higher frequency in another area [225]. Due to its involvement in top-down processing and the perceptual experience [226,227], alpha power has been demonstrated to be a promising index for the study of ASD-related difficulties. In addition, this index is highly sensitive to alterations in long-range connectivity [228,229]. Thus, integrating both frequencies using the alpha–gamma PAC could provide an accurate depiction of the local and global connectivity [196]. Several studies report altered alpha–gamma PAC among the ASD population [185,230,231], achieving a diagnostic accuracy of 90% [185]. However, while Berman and colleagues [231] found an increased alpha–gamma PAC within the ASD group, other studies [185,230] reported the opposite result. This discrepancy is likely due to differences in the experimental designs (resting state vs. visual task EEG recording) [185,230,231]. In general, as suggested by Kessler et al. [178], alterations in the alpha–gamma PAC may reflect an imbalance between excitatory/inhibitory activity in the perceptual brain network, resulting in a hypo- or hyper-sensitivity to various classes of stimuli and, thus, different clinical manifestations among the ASD population. This explanation could clarify the conflicting results (Table 4) about the alpha–gamma PAC, and then help to understand the nature of the perceptive alterations in individuals with ASD.

## 5. Attention Deficit Hyperactivity Disorder (ADHD)

ADHD is a neurodevelopmental disorder defined by two main pathological clusters [179]: (A) marked difficulties in the deployment and maintenance of the attentional focus (likely due to ineffective suppression of distracting stimuli) during most daily-life activities; (B) hyperactivity and poor impulsiveness control (i.e., logorrheic behavior, interrupting or intruding on others, blurting out answers before questions are over, squirming in seat). These two different core symptoms might occur separately or jointly, and have been associated with impairments in sensorimotor mechanisms, reward processing, affective self-regulation, and executive functions [232,233]. While the onset of such symptoms occurs before the age of 12, they tend to persist in adulthood, often in comorbidity with anxiety disorders, depression, or substance abuse [234,235].

### 5.1. The Role of Alpha Oscillations in ADHD’s Symtomatology

EEG analyses have played a pivotal role in the exploration of ADHD oscillatory biomarkers [43,46,47]. Although some findings might appear to be controversial due to the heterogeneity of ADHD phenotypes, existing literature suggests that individuals diagnosed with ADHD exhibit several alterations in oscillatory mechanisms crucial for cognition (see for instance [236]). Mid-frontal theta [237] and motoric beta alterations are often reported [238,239] together with impairments involving posterior alpha rhythms. In particular, it has been observed that the anticipation of visual distractors is linked to an alpha activity decrease over the visual cortex in typically developing individuals, whereas the anticipation of relevant stimuli increases it [30,49]. Moreover, an increase in the alpha power has been reported over relevant regions during high-demanding cognitive tasks, which is thought to suppress external inputs in order to support the relevant ones [49].

**Figure 4:**
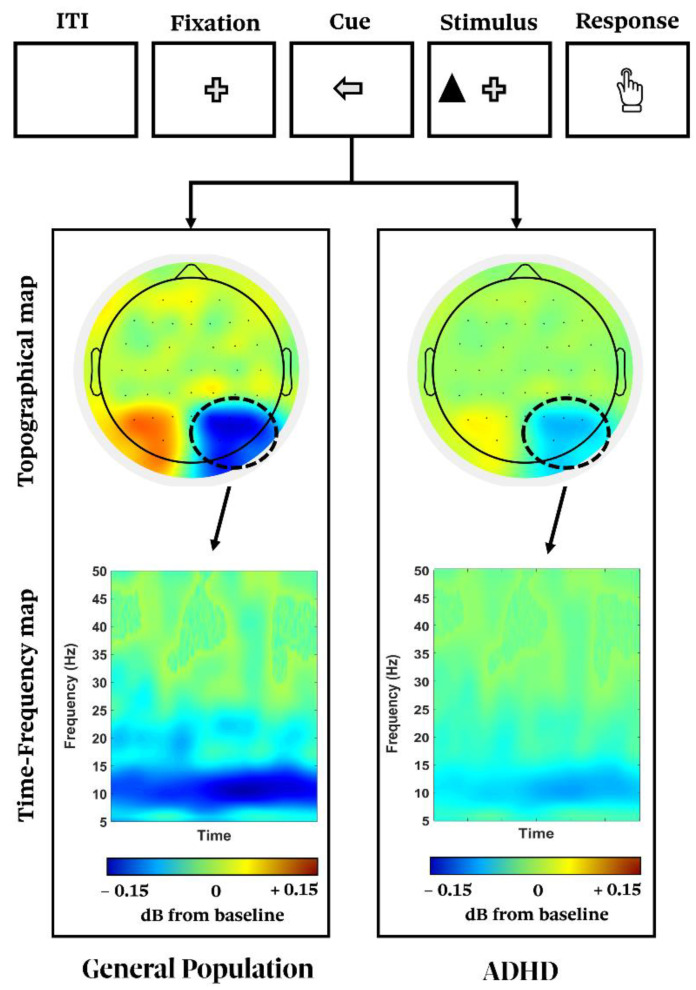
Simulated data showing a demeaned modulation in the inhibitory alpha power in ADHD (relative to controls) during the anticipation of a target stimulus in a visual task. Noteworthily, the lack of modulation comprises both contralateral ERD and ipsilateral ERS.

Consequently, ADHD’s symptoms may depend on abnormal oscillatory neural activity. Such alterations may also help to explain the WM deficit in ADHD. For instance, Lenartowicz and colleagues [240] recorded EEG activity in a large sample of ADHD children during a spatial WM task. EEG parameters associated with encoding, vigilance, and maintenance functions were analyzed. During the encoding, reduced occipital alpha power was reported, while the maintenance phase was accompanied by a greater power increase in the alpha band, interpreted as a compensatory response to weak alpha activity during the previous encoding stage. Such a failure in the encoding process was associated with poorer reading comprehension and executive functioning, as well with more severe ADHD symptomatology. Similarly, Hasler and colleagues [241] found reduced alpha and theta anticipatory activity in adults with ADHD when engaged in bottom-up and top-down attentive tasks. Such patterns were thought to reflect dysfunctional neural dynamics underlying the suppression of distractors and the prioritization of relevant information (Figure 4). The reduced ability to inhibit task-irrelevant stimuli may prompt or result from the enhanced tendency to mind-wander in ADHD. Some studies [48,242] linked alpha and theta reduction to spontaneous mind-wandering, namely the attentional shift from the task at hand to inner and unrelated thoughts [243]. The reduced ability to inhibit task-irrelevant stimuli may prompt or result from the enhanced tendency to mind-wander, and may trigger some of the core cognitive symptoms characterizing ADHD. In line with these considerations, Bozhilova and colleagues [242] asked adults with ADHD to perform a Go/No-Go task, while mind-wandering reports and EEG data (relative to both response execution and inhibition) were collected. The authors reported a higher error rate in the ADHD group and increased reaction time variability, along with reduced event-related alpha and beta suppression during No-Go trials. A hierarchical regression model applied to these measurements unveiled that ADHD diagnosis and proneness to mind-wandering might share a common oscillatory deficit, consistent with the notion that mind-wandering may be a supplemental pathological facet of this disorder. These pieces of evidence have been further supported by a later study [48], in which the authors reported how the reduction in the alpha and theta modulation in ADHD patients during WM and attentional paradigms is linked to mind-wandering episodes. Altogether, these findings suggest that alpha band alterations responsible for the impaired inhibition of task-irrelevant information might also underlie the increase in mind-wandering episodes.

Accordingly, a review by Lenartowicz and colleagues [49] highlighted how such attenuation in alpha suppression during visuo-attentional tasks is primarily linked to the ADHD inattentive profile, along with atypical lateralization patterns. Neurotypical individuals engaged in visuo-attentional paradigms show inter-hemispheric modulations in posterior alpha power. In particular, ERD is commonly reported in the hemisphere contralateral to the attended stimulus, while ERS is reported ipsilaterally. This inter-hemispheric imbalance is altered in ADHD patients, who do not display atypical modulations over posterior regions [244]. Similar results have been replicated by several different studies [245,246,247,248,249,250]. Specifically, Guo et al. [247] adopted a visuospatial attentional task in which the stimulus onset could be primed by a cue consisting of a gaze pointing toward either the left or right hemifield. The authors reported that, in the control group, an alpha lateralization with ERD was present in the hemisphere contralateral to the hemifield containing the to-be-attended upcoming stimuli, while children diagnosed with ADHD did not exhibit such a lateralization. This aberrant modulation was more pronounced in the left hemisphere, and was proven to correlate with both behavioral performance and severity of the inattentive symptoms. Interestingly, this lateralization pattern has been observed even over the sensorimotor areas when employing a motor task [248]. In this case, a reduction in the mu–alpha power over sensorimotor regions was reported to occur within the hemisphere contralateral to the hand performing the task in the neurotypical group, but not in ADHD. Moreover, in the ADHD group, a correlation was found between the aberrant lateralization in the oscillatory pattern and both the behavioral performance and difficulties to control disruptive motor activity and attentional processes in daily life.

Altogether, these results emphasize once more how the pathophysiological mechanisms triggering the symptoms of such a disorder might ensue from a deficient modulation of posterior alpha power, which may hinder the proper suppression of distracting information and, as a consequence, the allocation of attentional resources toward relevant stimuli. An interesting line of research, consequently, attempted to artificially modulate alpha activity with the aim of inducing shift in performance and in the symptoms’ severity in individuals with ADHD.

### 5.2. Normalizing Alpha Power Using the Neurofeedback Technique

As mentioned above [49,244,247], imbalanced alpha oscillations are an important biomarker of ADHD symptomatology. Accordingly, many authors used neurofeedback to normalize alpha power imbalances in ADHD patients. Neurofeedback-based protocols consist of training sessions where the participants learn to self-modulate their brain oscillations through real-time feedback, with the aim of concurrently reshaping specific behavioral routines [251,252]. The effectiveness of such approach in ADHD patients has been demonstrated by Escolano and colleagues [253], who were able to enhance fronto-midline upper alpha power in ADHD children undergoing 18 training sessions. The authors reported improvements in neuropsychological tests assessing WM, concentration, and impulsivity. Indeed, boosting alpha power appeared to generate a rebound effect that entailed a robust task-related alpha power decrease. A similar approach has been employed in another study [254] in which the authors induced a rebound effect through a neurofeedback protocol aimed at desynchronizing the alpha power during a Go/No-Go task. This resulted in a subsequent power normalization, and in improvements in terms of motor inhibition. Furthermore, results highlighting the relevance of alpha desynchronization in attentive visual paradigms [49] have been adopted to reduce the power in this frequency range via neurofeedback protocols in ADHD individuals, prompting significant improvements to sustained attention [255].

In conclusion, the literature revealed how some electrophysiological features such as the posterior alpha suppression seem to be linked to the occurrence of ADHD symptomatology [49,51,240,244]. These findings are further strengthened by neurofeedback studies, which highlighted how normalizing these aberrant oscillatory patterns could benefit ADHD patients. Altogether, these pieces of evidence (Table 5) underline the benefits of looking into oscillatory activity in the alpha band, as well as how this kind of information can be used to induce an amelioration.

## 6. General Conclusions

The present work aimed to review the last 10 years of research on the role of alpha oscillations among the main neuropsychiatric disorders, such as SSD, MDD, ASD, and ADHD, to summarize the high volume of publications on these topics.

The main findings suggest that individuals with SSD may display, in task-positive regions, a reduction in both the alpha power and frequency compared to healthy individuals, while in task-negative areas, the oscillatory power appears to be increased [76,80]. Further, FAA has proven to be a valuable proxy of MDD emergence, since these patients seem to display an increase in the alpha power over the left frontal hemisphere, and a decrease in the right one compared to controls [118,131]. In individuals with ASD, ground evidence suggests the presence of an imbalance in the alpha band’s neural connectivity, with a local increase in short-range connectivity in both the posterior and anterior networks, while the long-range connectivity between these two regions is reduced [180,181,194]. Lastly, a lower modulation of contralateral alpha ERD and ipsilateral alpha ERS has been found in ADHD during the anticipation of target stimuli in a visual task, when compared to typically developing individuals [48,49].

Nonetheless, robust electrophysiological biomarkers of neuropsychiatric disorders are still difficult to identify. This seems to be due to the enormous differences in the clinical conditions and their behavioral manifestations. Furthermore, in the studies we considered, several methodological discrepancies emerged. In particular, some studies use high-density EEG while others do not, resulting in a range between 16 and 256 electrodes, and, similarly, important discrepancies can be found regarding the number of individuals included in the studies. Other relevant discrepancies concern the participants’ age (children vs. adults), the disorder onset (first or not), whether the person is under treatment (behavioral/pharmacological), or the choice to record the EEG signal during a task or in a resting state condition. Moreover, a consistent heterogeneity can be seen in the biotypes included in the research (for instance, considering the inattentive, the hyperactive, or both subtypes in the ADHD sample). Altogether, these factors might lead to discrepancies in the scientific literature, despite searching for the same condition. Thus, even the line of research focusing specifically on the role of alpha bands found conflicting results with small differences in the experimental methodology.

The current work also highlights how the availability of several EEG indices to address the same issue allows for a deeper understanding of certain specific aspects of the phenomena. Indeed, most of these measures are often based on the investigation of different alpha parameters, not allowing for a direct comparison between studies. This is often regarded as controversial evidence, making it difficult to draw parallels even with regard to studies sharing methodological similarities. Therefore, herein, we emphasize the importance for future research to adopt a standardized methodological procedure, allowing for a better comparison of these fragmented pieces of evidence. Such an approach might also help provide an overall conclusion regarding the magnitude of the effects found herein. This may strengthen our highlights, allowing researchers and clinicians to use the aforementioned finding to plan more effective evidence-based treatments tailored to each specific clinical phenotype. In this paper, we attempted to circumscribe these conditions and their electrophysiological peculiarities, for example by excluding from the current work studies investigating comorbidities. Forthcoming studies should fill this gap, since tracing links between these pathological conditions would provide a more exhaustive picture, possibly illustrating how these behavioral and neurophysiological features overlap. This would also help us to understand which alpha-based indices better explain the commonalities between these disorders.

In conclusion, the last 10 years of research have brought several proofs on the role of alpha oscillations in neuropsychiatric disorders. In particular, alterations in the alpha power and frequency have been reported in patients with SSD [76,80], whereas FAA has been proven to be able to discriminate MDD from healthy controls [118,131]. Furthermore, an imbalance in the alpha band brain connectivity between long- and short-range regions has been observed in ASD [180,181,194]. Finally, ADHD seems to display reduced alpha power during stimuli processing relative to controls [48,49].

Hence, the alpha band indices may represent reliable and practical measures to support the clinician during the diagnosis formulation, the choice and the evaluation of a treatment, or the assessment of the symptoms.

## Figures and Tables

**Table 1:** Table showing the main electrophysiological indices used for the review, along with a brief description.

Table of EEG Indices		
Index	Full Name	Description
ERD/ERS	event-related synchronization/desynchronization	Amplitude up/down-modulation in response to a specific event, due to a synchronized activity of a large number of neurons
FAA (PAA)	frontal (or posterior) alpha asymmetry	Relatively higher alpha power recorded from the left (as compared to the right) hemisphere over frontal (or posterior) regions
IAF	individual alpha frequency	The frequency bin displaying the highest power value within the alpha band (8–13 Hz)
ITC	intertrial coherence	The degree of oscillatory phase-synchronization across different trials
PAC	phase amplitude coupling	Coupling between the phase of slower oscillations with the amplitude of faster oscillations (i.e., reflecting integrative mechanisms of neural activity within the brain)
PSD	power spectral density	Measure of signal’s power content versus frequency

**Table 2:** Table representing the main electrophysiological findings in SSD, with the methods and the studies who contributed to them.

Schizophrenia Spectrum Disorder (SSD)		
Studies	Analytic Method	Main Findings
[68,69,70,73,77,80]	Resting state IAF	Slower IAF over posterior regions
[60,61,62,63,64,65,66]	Resting state PSD	Posterior and frontal Alpha power reduction
[75,76,77]	Resting state PSD	Alpha power increase in the DMN
[68,75,76,82,83,84,85,86,87,88,97,109,114,117]	Functional connectivity	Aberrant long-range functional connectivity
[71,93,94,96,99,100,101,102,103,104,105]	Auditory evoked response	Aberrant Alpha ERD/ERS over posterior and frontocentral areas
[106,107,108,110,111]	Evoked response during WM and attentive tasks	Aberrant Alpha ERD modulation

**Table 3:** Table representing the main electrophysiological findings in the MDD, with the methods and the studies who contributed to them.

Major Depressive Disorder (MDD)		
Studies	Analytic Method	Main Findings
[118,128,129,130,131,132,133,134,135,136,137]	Resting state PSD	Frontal Alpha asymmetry
[118,131,141,142]	Resting state PSD	Posterior Alpha asymmetry
[144,145,146,149,150,151,152]	Resting state PSD	Aberrant posterior Alpha power
[126,127,156,157,158,160,161,162,163,164,169]	Functional connectivity	Aberrant short and long-range functional connectivity
[134,167,168,170]	Evoked response during WM and attentive tasks	Aberrant Alpha power over posterior (reduced) and midfrontal (increased) areas

**Table 4:** Table representing the main electrophysiological findings in the ASD, with the methods and the studies who contributed to them.

Autistic Spectrum Disorder (ASD)		
Studies	Analytic Method	Main Findings
[195,197,198]	Task-induced PSD	Impaired modulation of Alpha power
[186,203,215]	Topographical distribution of Alpha power	Aberrant Alpha power in frontal regions
[185,230,231]	PAC	Aberrant Alpha–Gamma PAC
[180,181,184,185,187,189]	Functional connectivity	Aberrant short- (enhanced) and long-range (reduced) functional connectivity
[201,202]	Evoked response during motor tasks	Less suppressed Mu–Alpha ERD/ERS over sensorimotor areas

**Table 5:** Table representing the main electrophysiological findings in ADHD, with the methods and the studies who contributed to them.

Attention Deficit Hyperactivity Disorder (ADHD)		
Studies	Analytic Method	Main Findings
[49,240,241,244,246,247,250]	Topographical distribution of Alpha power during attentional tasks	Aberrant Alpha ERD/ERS lateralization
[240,245]	Topographical distribution of Alpha power during motor tasks	Reduced Mu–Alpha ERD/ERS lateralization
[48,242]	Evoked response during spontaneous mind wandering and tasks	Reduced Alpha ERD/ERS

## Abbreviations

ACC	Anterior cingulate cortex
ADHD	attention deficit hyperactivity disorder
ASD	autism spectrum disorders
dlPFC	dorsolateral prefrontal cortex
DMN	default mode network
DSM-5	diagnostic and statistical manual of mental disorders
EEG	electroencephalogram
ERD	event-related desynchronization
ERP	event related potential
ERS	event-related synchronization
FAA	frontal alpha asymmetry
IAF	individual alpha frequency
ITC	intertrial coherence
MDD	major depressive disorders
MEG	magnetoencephalography
mPFC	medial prefrontal cortex
OCC	occipital cortex
PAA	posterior/parietal alpha asymmetry
PAC	phase amplitude coupling
PCC	posterior cingulate cortex
PFC	prefrontal cortex
PPC	posterior parietal cortex
PSD	power spectral density
SSD	schizophrenia spectrum disorder
tACS	transcranial alternating current stimulation
tDCS	transcranial direct-current stimulation
TMS	transcranial magnetic stimulation
WM	working memory
